# Doctors' War Bonus Increased

**Published:** 1919-06-28

**Authors:** 


					Doctors' War Bonus Increased,
May, Major 4stor ^Chaimiir^nf tIle Medical Association to the Insurance Commission in the middle of
;ndyLs*t? Committee, has consulted the Treasu^
a payment of war bonus to Insurance practitioners ? the Gov??n?ent prepared to make, for the year 1919,
r-.. 1010 / a substantially enhanced rate over and above the rate paid
for the year 1918.

				

